# Validation of Nursing Outcome Indicators in Patients with Postsurgical Delirium

**DOI:** 10.17533/udea.iee.v41n3e11

**Published:** 2023-10-26

**Authors:** Estela Melguizo-Herrera, Yolima Manrique-Anaya, Claudia Torres-Contreras, Raquel Rivera-Carvajal, Cesar Hueso-Montoro

**Affiliations:** 1 Nurse, PhD. Professor. Email: emelguizoh@unicartagena.edu.co Universidad de Cartagena Colombia emelguizoh@unicartagena.edu.co; 2 Nurse, Masters. Professor. Email: ymanriquea@unicartagena.edu.co Universidad de Cartagena Colombia ymanriquea@unicartagena.edu.co; 3 Nurse, PhD. Professor. Email: clau.torres@mail.udes.edu.co Universidad de Santander Colombia clau.torres@mail.udes.edu.co; 4 . Nurse, Masters. Professor. Email: raq.rivera@mail.udes.edu.co Universidad de Santander Colombia raq.rivera@mail.udes.edu.co; 5 Nurse, PhD. Professor, Universidad de Jaén, Spain. Email: chueso@ujaen.es, corresponding author Universidad de Jaén Universidad de Jaén Spain chueso@ujaen.es; 6 Universidad de Cartagena. Cartagena, Colombia. Universidad de Cartagena Universidad de Cartagena Cartagena Colombia; 7 Faculty of Medical and Health Sciences, Universidad de Santander. Bucaramanga, Colombia. Universidad de Santander Faculty of Medical and Health Sciences Universidad de Santander Bucaramanga Colombia

**Keywords:** validation study, delirium, adults, standardized nursing terminology, critical care units, estudio de validación, delirio, adulto, terminología normalizada de enfermería, unidades de cuidados intensivos, estudo de validação, delírio, adulto, terminologia padronizada em enfermagem, unidades de terapia intensiva

## Abstract

**Objective.:**

To validate the content of the indicators proposed from the Nursing Outcome Classification in a care plan for delirium management in older adults.

**Methods.:**

Content validity study, conducted under the expert judgment technique. The procedure was developed in five moments: organization of indicators that respond to the nursing outcome classification for delirium management, support with literature of the indicators that responds to the result, selection of experts, establishment of agreements, and discussion. Quality criteria evaluated: pertinence and relevance, the Content Validity Coefficient and average scores assigned by the experts were calculated.

**Results.:**

The study had the participation of 14 experts. The indicators, according to criteria of pertinence and relevance evaluated by experts showed a global average content index value of 0.93; 97.05% (66) of the indicators had Content Validity Coefficient > 0.75.

**Conclusion.:**

The quantitative findings of the indicator validation process showed high relevance and pertinence index, which favors their being applied to measure care changes in patients with delirium.

## Resumen

**Objetivo.:**

Validar el contenido de los indicadores propuestos a partir de *Nursing Outcome Classification* en un plan de cuidados para el manejo de delirium en los adultos mayores.

**Métodos.:**

Estudio de validación de contenido realizado bajo la técnica de juicio de expertos. El procedimiento se desarrolló en cinco momentos: organización de los indicadores que responden a la clasificación de resultados de enfermería para manejo de delirium, soporte con literatura de los indicadores que responde al resultado, selección de expertos, establecimiento de acuerdos y discusión. Criterios de calidad evaluados: pertinencia y relevancia. Se calculó el Coeficiente de Validez de Contenido, así como los promedios de los puntajes asignados por los expertos.

**Resultados.:**

Se contó con la participación de 14 expertos. Los indicadores según los criterios de pertinencia y relevancia evaluados por expertos mostraron un valor global promedio de índice de contenido de 0.93. El 97.05% (66) de los indicadores presentaron Coeficiente de Validez de Contenido mayor a 0.75.

**Conclusión.:**

Los hallazgos cuantitativos del proceso de validación de los indicadores mostraron alto índice de relevancia y pertinencia lo que favorece que puedan ser aplicados para medir cambios de cuidado en los pacientes con delirium.

## Resumo

**Objetivo.:**

Validar o conteúdo dos indicadores propostos pela Classificação dos Resultados de Enfermagem em um plano de cuidados para o manejo do delirium em idosos.

**Métodos.:**

Estudo de validação de conteúdo, realizado através da técnica de julgamento de especialistas. O procedimento foi desenvolvido em 5 momentos: organização dos indicadores que respondem à classificação dos resultados de enfermagem para manejo do delirium, suporte com literatura dos indicadores que respondem ao resultado, seleção de especialistas, estabelecimento de acordos e discussão. Foram calculados os critérios de qualidade avaliados: relevância e pertinência, o Coeficiente de Validade de Conteúdo e as médias das notas atribuídas pelos especialistas.

**Resultados.:**

Participaram 14 especialistas. Os indicadores segundo os critérios de relevância e pertinência avaliados por especialistas apresentaram valor médio do índice de conteúdo global de 0.93. O 97.05% (66) dos indicadores apresentaram Coeficiente de Validade de Conteúdo superior a 0.75.

**Conclusão.:**

Os achados quantitativos do processo de validação dos indicadores apresentaram alto índice de relevância e pertinência, o que favorece sua aplicação para mensurar mudanças no cuidado de pacientes com delirium.

## Introduction

The nursing care process (NCP) is a tool that allows professional nurses to apply the scientific method to provide care in continuous and individualized manner, rationally, logically, and systematically.^1^ Application of the NCP has been enriched with the use of the nursing taxonomies about results^2^ and interventions^3^ that summarize the diagnoses of the North American Nursing Diagnosis Association (NANDA).^4^ The use of nursing outcomes from the Nursing Outcome Classification (NOC) began in the 1960s; its objective was to assess the quality of nursing care. From there, processes of adjustments and measures have been developed. Currently, the NOC classification, in the sixth-edition text, contains 540 results, 7 domains, and 32 classes. The NOC results, in care plans, quantify the state, behavior or perception of the patient expected to occur in specific moments of a care episode. Each NOC has a battery of indicators evaluated by the professional. The scores issued from the NOC, using a Likert scale from one to five, measure the initial and final state of the diagnosis intervened and, consequently, the change in the patient's health, if any.^2^ However, for the NOC to be replicated, it is necessary to validate its indicators, which analyze the adequate psychometric characteristics to measure that for which it was designed.^5^ This content validity consists in the evaluation by experts on the study theme of the relevance (importance of the item measured in the context) and pertinence (quality of the item of corresponding entirely to the context) of the items included in a scale.^6^^,^^7^

The evaluation of care by nursing professionals to patients with delirium is part of interdisciplinary work, given that management of the diagnosis is multicausal, commonly goes unnoticed by over 67% of non-psychiatric physicians^8^^,^^9^ and only 22% of the nursing staff identifies delirium and its criteria.^10^ A study conducted in Cartagena (Colombia) affirmed that 54.23% of medical professionals and nurses evaluate delirium occasionally, 68.31% evaluate delirium through general clinical evaluation; moreover, 52.11% of the professionals consider that delirium is a pathology that requires intervention.^11^

The foregoing identifies a void in the continuity of caring for delirium and in unifying the results and interventions to implement. In addition, it has led to the need to use evaluation scales for the early detection and management of delirium. However, few of these scales or tools have been validated and are typical of nursing professionals.^12^ Based on the aforementioned, the aim of this study was to validate the indicators contained in the NOC of a nursing care plan developed previously by the research team to manage elderly patients with postsurgical delirium. This care plan included 11 NOC: cognition, nutrition, cardiac pump effectiveness, circulatory state, hydric balance, respiratory state, pain control, mobility, electrolyte and acid-base balance, sleep and vital signs.^8^ The vital sign NOC was identified as an activity inherent to the care provided by professionals in addition to being in the protocols of hospital institutions, hence, it was excluded from the validation. The context to which the care plan is applied corresponds to the intensive care units (ICU) and hospitalization units where postsurgical delirium is treated.

## Methods

*Design.* A content validity process was carried out by expert judgment using the Delphi method.^13^ The population was comprised by nurses with graduate studies, experience in ICU and hospitalization of at least two years, including management of patients with delirium and the nursing care process. The participants were recruited according to their compliance with the inclusion criteria through contact with the academic units linked to the hospital institutions participating in the research. The sample was taken according with the criteria by Hyrkäs *et al.,*^14^ who manifest that 10 experts provide a reliable estimation of the content validity of an instrument; in turn, Voutilainen & Liukkonen^15^ indicate that if 80% of the experts have agreed with the validity of an item, it can be incorporated onto the instrument.

*Data collection.* The study was conducted during two moments: the first from January to May of 2021, with the literature search by the researchers to select the indicators of the results that have been used to evaluate the evolution of patients with delirium, according to NANDA diagnoses established in a prior study carried out in the city of Cartagena (Colombia).^8^ The second moment was the evaluation by nurses from August to October of 2021, with the participation of 14 experts from four hospital institutions who assessed the pertinence and relevance of the NOC indicators with the aid of a form delivered via e-mail. The nurses took between five and twenty days to respond.

*Instruments.* The form used contained two questionnaires: one for sociodemographic variables (age, academic training, work experience, knowledge about the NCP, experience in managing patients with delirium) and another that contained the results to be evaluated with their indicators according to relevance and relevance. Each indicator corresponded to an item from the form, so that nurses made their judgment based on the relevance and pertinence measured with a Likert scale, where they assigned a score from one to five, considering one as not relevant or not pertinent and five as very relevant or pertinent.^16^

*Data analysis.* To analyze the information, relative and absolute frequencies were calculated for categorical or qualitative variables and measures of central tendency and dispersion for numerical variables. The Content Validity Coefficient (CVC) was calculated for each of the items. When the experts assigned scores of 4 or 5 (relevant and pertinent), these were assumed as a favorable concept and the fraction was calculated with the total responses for each item, following what was proposed by Polit *et al*.,^16^ in addition, the mean of the scores of the indicators is presented. Calculations were performed on the STATAv statistical software.

*Ethical considerations.* The study was based on Resolution 8430 of 1993 by the Colombian Ministry of Health^17^ and the Declaration of Helsinki promulgated by the World Medical Association in 2000.^18^ The research was considered a study without risk. Among the ethical principles of the research, autonomy was taken into account. The informed consent was requested to be signed and forwarded by e-mail; the objective of the research was explained to the participants in the document, highlighting that their participation and withdrawal was voluntary. The study was endorsed by the ethics committee at Universidad de Cartagena (Colombia) with minute 02 of May 11, 2021.

## Results

Fourteen expert judges participated by reviewing the indicators from 10 NOC. The experts were characterized, thus: eight with Masters educational level and 7 with specialization; with an average of 15.28 **±** 8.87 years of work experience and an average of 9.21 **±** 4.26 years managing patients with delirium (Table 1).

**Table 1:** Description of participating expert judges

Participant	Sex	Educational level	Work experience	Years managing patients with delirium
P1	Woman	Specialist	8	6
P2	Woman	Masters	20	10
P3	Woman	Masters	25	10
P4	Woman	Specialist	5	5
P5	Woman	Masters	15	10
P6	Woman	Masters	16	5
P7	Woman	Specialist	8	7
P8	Woman	Specialist	2	2
P9	Man	Masters	22	16
P10	Man	Masters	21	10
P11	Woman	Masters	26	15
P12	Woman	Masters	30	16
P13	Man	Specialist	7	7
P14	Woman	Specialist	10	10

Table 2 shows the CVC according to the pertinence and relevance criteria of each of the NOC indicators; 68 indicators were linked, among which 9 from cognition NOC, 7 from sleep, 10 from nutrition, 7 from cardiac pump effectiveness, 7 from circulatory state, 5 from hydric balance, 10 from electrolyte and acid-base balance, 6 from respiratory state, 5 from pain control, and 7 from mobility. It is noted that 85.52% (58) had CVC > 0.80, 97.70% (66) with CVC > 0.75, and only two items had low scores, which are “040004 Ejection fraction” with CVC = 0.64 and “040105 Central venous pressure” with CVC = 0.57.

**Table 2:** Content Validity Coefficient and average score of the NOC indicators

Indicators	Pertinence 181.		Relevance 183.		Total 185. 186.		
	CVC	Average	CVC	Average	CVC	Average
Cognition						
90003 Aware	1.00	4.50	0.85	4.50	0.93	4.50
90004 Concentrates	1.00	4.57	1.00	4.50	1.00	4.54
90005 Is oriented	1.00	4.78	0.78	4.85	0.89	4.82
90006 Immediate memory	0.78	4.14	1.00	4.35	0.89	4.25
90007 Recent memory	0.78	4.07	1.00	4.50	0.89	4.29
90009 Processes information	0.92	4.50	1.00	4.57	0.96	4.54
90013 Understands meaning of situations	0.85	4.42	0.85	4.42	0.85	4.42
90014 Clear communication according to age	0.85	4.28	0.78	4.14	0.82	4.21
90015 Adequate communication according to age	1.00	4.71	0.92	4.57	0.96	4.64
Sleep 277. 278. 279. 280.						
000401 Hours of sleep	1.00	4.87	1.00	4.92	1.00	4.90
000404 Quality of sleep	1.00	4.78	1.00	4.92	1.00	4.85
000418 Sleeps entire night	0.92	4.50	0.78	4.35	0.85	4.43
000420 Comfortable room temperature	1.00	4.64	0.92	4.64	0.96	4.64
000421 Difficulty falling asleep	0.92	4.71	0.92	4.57	0.92	4.64
000406 Interrupted sleep	0.92	4.57	0.85	4.57	0.89	4.57
060014 Blood urea nitrogen	0.78	4.14	0.78	4.07	0.78	4.11
Nutrition						
100901 Calorie intake	0.85	4.28	0.85	4.35	0.85	4.32
100902 Protein intake	0.92	4.50	0.85	4.64	0.89	4.57
100903 Fat intake	0.92	4.50	0.85	4.57	0.89	4.54
100904 Carbohydrate intake	0.92	4.50	0.85	4.57	0.89	4.54
100910 Fiber intake	0.92	4.50	0.85	4.57	0.89	4.54
100905 Vitamin intake	0.85	4.28	0.78	4.35	0.82	4.32
100906 Mineral intake	0.78	4.00	0.78	4.07	0.78	4.04
100907 Iron intake	0.78	4.21	0.71	4.21	0.75	4.21
100908 Calcium intake	0.85	4.42	0.78	4.50	0.82	4.46
100911 Sodium intake	0.92	4.50	0.85	4.50	0.89	4.50
Cardiac pump effectiveness						
040001 Systolic blood pressure	1.00	4.92	1.00	4.92	1.00	4.92
040019 Diastolic blood pressure	1.00	4.92	1.00	4.92	1.00	4.92
040002 Heart rate	1.00	5	1.00	4.92	1.00	4.96
040003 Cardiac index	1.00	4.78	1.00	4.57	1.00	4.68
040004 Ejection fraction	0.64	4	0.64	3.92	0.64	3.96
040020 Urinary output	0.92	4.71	0.85	4.5	0.89	4.61
040025 Central venous pressure	0.71	4.07	0.78	4.35	0.75	4.21
Circulatory state						
040101 Systolic blood pressure	0.92	4.64	0.92	4.57	0.92	4.61
040102 Diastolic blood pressure	0.92	4.64	0.92	4.57	0.92	4.61
040104 Mean arterial pressure	1.00	4.92	1.00	4.85	1.00	4.89
040105 Central venous pressure	0.57	4.64	0.57	4	0.57	4.32
040135 Partial pressure of oxygen in blood	0.85	4.64	0.85	4.57	0.85	4.61
040137 Oxygen saturation	0.92	4.64	0.85	4.57	0.89	4.61
040140 Urinary output	0.92	4.50	0.85	4.42	0.89	4.46
Hydric balance						
60107 Balanced daily inputs and outputs	1.00	4.78	1.00	4.71	1.00	4.75
60116 Cutaneous hydration	0.85	4.5	0.92	4.50	0.89	4.50
60118 Serum electrolytes	1.00	4.92	1.00	4.85	1.00	4.89
60119 Hematocrit	1.00	4.85	1.00	4.78	1.00	4.82
60127 Amount of urine	0.92	4.71	0.92	4.57	0.92	4.64
Electrolyte and acid-base balance						
060003 Breathing frequency	0.92	4.64	0.92	4.71	0.92	4.68
060005 Serum sodium	0.92	4.64	0.92	4.57	0.92	4.61
060006 Serum potassium	0.92	4.57	0.92	4.57	0.92	4.57
060007 Serum chloride	0.85	4.50	0.92	4.57	0.89	4.54
060008 Serum calcium	0.78	4.28	0.71	4.28	0.75	4.28
060009 Serum magnesium	0.78	4.92	0.71	4.28	0.75	4.60
060010 Serum pH	1.00	4.92	1.00	4.92	1.00	4.92
060013 Serum bicarbonate	1.00	4.78	1.00	4.92	1.00	4.85
060026 Serum glucose	1.00	4.78	0.85	4.71	0.93	4.75
060014 Blood urea nitrogen	1.00	4.78	0.92	4.64	0.96	4.71
Respiratory state						
41004 Respiratory frequency	1.00	4.92	1.00	5	1.00	4.96
41005 Respiratory rate	1.00	4.92	1.00	5	1.00	4.96
41007 Pathological respiratory sounds	1.00	4.92	1.00	5	1.00	4.96
41012 Ability to eliminate secretions	1.00	4.71	1.00	4.78	1.00	4.75
41017 Depth of inspiration	1.00	4.78	1.00	5	1.00	4.89
41018 Use of accessory muscles	1.00	4.92	1.00	4.92	1.00	4.92
Pain control						
160502 Recognizes beginning of pain	0.92	4.78	1.00	4.71	0.96	4.75
160509 Recognizes symptoms associated with pain	1.00	4.85	1.00	4.71	1.00	4.78
160511 Reports controlled pain	1.00	4.71	0.92	4.57	0.96	4.64
160513 Reports changes of symptoms to health staff	1.00	4.64	1.00	4.57	1.00	4.61
160516 Describes pain	1.00	4.85	1.00	4.78	1.00	4.82
Mobility						
020801 Maintaining balance	0.85	4.35	0.92	4.57	0.89	4.46
020809 Coordination	1.00	4.57	0.92	4.57	0.96	4.57
020815 Bone integrity of the lower extremity	0.85	3.92	0.71	4	0.78	3.96
020803 Muscular motion	1.00	4.71	1.00	4.71	1.00	4.71
020804 Articular motion	0.85	4.28	0.85	4.28	0.85	4.28
0200802 Maintaining body position	0.92	4.64	1.00	4.78	0.96	4.71
020806 Ambulation	0.92	4.35	0.92	4.42	0.92	4.39

Table 2 shows that the health physiology domain contains the most nursing outcomes (NOC) 7/10; in these seven results, each indicator was scored by the experts with agreement among them > 4.20, corresponding to being pertinent and relevant. Likewise, it is possible to identify the relationship between results, like Cardiac pump effectiveness and Circulatory state, where indicators to be evaluated are shared, and between Hydric balance and Electrolyte and acid-base balance, which share indicators to measure. It is shown how the nutritional state NOC is a necessary element to be incorporated in monitoring of patients with eight indicators. 

## Discussion

The findings reveal that the indicators selected for each of the 10 NOC contemplated in this study are pertinent and relevant, which is why they can be considered by nursing professionals to evaluate the evolution of care for adult and elderly patients with postsurgical delirium. Most of the indicators had a CVC mean > 0.80. The evolution is complemented with monitoring of vital signs.

This work is among the first to use NOC indicators to measure the evolution of patients with postsurgical delirium, a fact that also hinders discussion with other previous studies. The expert participants showed graduate academic level, which leads to believe that they managed adequately the concept of patients with delirium. It is noteworthy that the use of these indicators may complement the use of other evaluation instruments, highlighting the CAM-ICU scale, considered the valid and reliable psychometric instrument to issue the delirium diagnosis,^19^^,^^20^ as well as to determine the prevalence of the problem.^21^

In relation with the indicators, the NOC result: Cognition showed nine indicators to measure, with “is oriented” being relevant. Thus, is how Inouye *et al*.,^22^ showed in their study that specific interventions of information on orientation, for example, date, time, names of hospital staff, cognitive stimulation activities, among others, reduce significantly the number and duration of delirium episodes in relation with patients not monitored with the information described. Due to being quite close to patients, nurses are perhaps the individuals who can better stimulate patient orientation in the ICU and contribute to earlier reversal of delirium. 

Moreover, from the nutrition NOC, it was evidenced that protein, fat, carbohydrate, fiber, and calcium intake is considered important by the experts. The literature shows that managing elements, like electrolytes and proteins is key. It has been demonstrated that biochemical alterations (low sodium and potassium levels) and a low body mass index (BMI) are indicators that lead to deteriorated cognitive function that predispose the severity of delirium.^23^ Thereby, the need is evidenced for biochemical monitoring in coordination with other disciplines. 

The NOC indicators related with cardiac pump effectiveness and circulatory state relate to monitoring of diastolic and systolic blood pressure in patients, as mechanism to control adequate and sufficient perfusion at the cerebral level in these patients. This is coherent with the findings by Wang *et al*.,^24^ who reported greater prevalence of postsurgical delirium in patients undergoing surgical procedures that occurred with low saturations due to their decreased mean arterial pressure.

It was evidenced in the results that maintaining electrolytes, pH, bicarbonate, and hematocrits are necessary variables to measure during the patient’s clinical signs and symptoms. This agrees with that explained by Artola *et al.,* ^(^^25^ who reported that early detection of dehydration signs, states of oliguria added to maintaining a hydric balance lead to early identification of hydro-electrolyte alterations and, with this, permit unifying correct treatment actions by the interdisciplinary team.

With regards to the respiratory state NOC, the experts classified the six indicators with maximum pertinence and relevance, which could infer that oxygenation is an essential process for optimal brain functioning, thought processes and the state of cognition that, in situations like surgical procedures can be compromised, causing delirium in patients. Therefore, if each of the indicators is evaluated in time, the condition could be controlled early. In this respect, Recasens *et al.,*^26^ through a program, via prior training and formation of nursing professionals on the management of the NANDA, NOC, NIC taxonomy, managed to incorporate the evaluation of the respiratory state NOC and, with this, evaluation of care was standardized, in addition to supporting nursing professionals to pay attention to changes specific to the respiratory state.

Finally, upon analyzing the mobility, pain control and sleep NOC indicators, it may be documented that their articulation is important in the evolution of the delirium condition; patients can recognize the moment when their pain begins and mention these changes to the health professional, they could correlate whether it is during mobility, exercise, or postural change, in addition to the fact that it could cause changes in the hours and quality of sleep of patients with delirium. The foregoing is corroborated with the study by Rodríguez *et al*.,^27^ where the range of pain prevailing in postsurgical patients was slight, attributed, among other factors, to its early detection and to correcting activities, like early mobilization to ensure that the patient intervened reconciles sleep.

Among the strengths of this study, it is considered that the indicators evaluated guide in care process for patients with delirium in ICU and hospitalization contexts in which this condition can develop. It is convenient to continue exploring other interventions that involve, for example, the family,^28^ or that take place in other services, like emergency,^29^ besides analyzing the effect on the workload^30^ of the nursing staff given the occurrence of delirium in patients. Further, it would be worth investigating possible interventions or guidelines at the home level,^31^ as well as performing post-discharge research to determine the quality of life and functionality ^(^^32^ in patients who have endured this event.

Among the limitations of this study, it should be noted that the validation was applied by experts who manage the taxonomies, which is why its implementation requires extensive knowledge by all nursing professionals to manage a clinical diagnosis, like delirium. Furthermore, it is necessary to continue advancing in validation studies involving more countries and which explore more advanced procedures.

Regarding the practical implications of this research, it is worth highlighting that application of the NANDA, NOC, NIC taxonomy allows obtaining precise information that should be assumed by health professionals, in their care practice, because it has been proven that it allows them to make their practice fluid, define interventions patients require, and evaluate the evolution of the diagnosis labeled. In line with the aforementioned, this requires training on managing taxonomies, something that should be evaluated in undergraduate and graduate training.

Conclusion. The findings, herein, show a high relevance and pertinence index of the indicators evaluated, which favors their being applied to measure care changes in patients with delirium. Among these changes, indicators of aspects related to sleep quality, biochemical measures, pain control or cognition are included.
